# Study on vibration characteristics of shearer spiral drum and fatigue life prediction of cutting unit housing under multi-factor coupling

**DOI:** 10.1371/journal.pone.0352564

**Published:** 2026-09-09

**Authors:** Yadong Wang, Baoxuan Jia, Chuang Ge, Guocong Lin, Lijuan Zhao, Jiagui Yu, Xunan Liu

**Affiliations:** 1 School of Mechanical Engineering, Liaoning Technical University, Fuxin, China; 2 Liaoning Province Large Scale Industrial and Mining Equipment Key Laboratory, Fuxin, China; King Mongkut's University of Technology North Bangkok, THAILAND

## Abstract

Severe vibration and fatigue damage of the cutting unit housing remain key problems in thin-coal-seam shearers because coal-rock heterogeneity, drum structure, and motion parameters jointly generate nonlinear, time-varying cutting loads. Because field tests cannot efficiently isolate these coupled effects or reproduce long-term loading, a validated numerical workflow is needed for vibration-control design and preliminary fatigue assessment. This study develops a DEM-MFBD bidirectional rigid-flexible coupling model of the MG2 × 55/250-BW shearer using EDEM, Pro/E, and RecurDyn. Cutting tests validate the drum vibration response, and a Taguchi L18(3^4^ × 2^1^) mixed-level design evaluates gangue hardness, blade spiral angle, traction speed, drum speed, and pick arrangement. Fixed-effects ANOVA shows that gangue hardness is dominant, contributing 81.68%−82.03% of the variation in the maximum, minimum, and RMS responses; traction speed and pick arrangement are secondary. Increasing gangue hardness raises the peak and RMS vibration accelerations by approximately 35%, whereas the cross-type pick arrangement reduces them by 7.73% and 8.57%, respectively. The validated load spectrum is then combined with rainflow counting, the Palmgren-Miner rule, and RecurDyn's stress-based Manson-Coffin criterion, to identify fatigue-critical housing regions. Weak regions occur at the lug transition, drum connection, and gear-shaft holes.Under the worst working condition, the cycle number corresponding to the node with the minimum fatigue life of the housing is $3.5053\times 10^{10}$, which meets the service requirements. The workflow links cutting-load generation, vibration response, factor sensitivity, and model-based fatigue screening. The research lays a theoretical foundation and provides technical support for the structural optimization of shearer helical drums, operating parameter matching, and reliability design of cutting unit housings, which is of great significance for improving the operational efficiency and safety of coal shearers.

## Introduction

The cutting and fragmentation of coal and rock by a shearer is a complex dynamic process influenced by multiple interacting factors [1–3]. These factors include the physical and mechanical properties of the coal seam, the coupling interaction between the spiral drum and the coal-rock interface, the structural parameters of the drum, and the kinematic parameters of the shearer. The loads experienced by the spiral drum and cutting components exhibit nonlinear, time-varying characteristics, which directly affect vibration and deformation. Intense vibration can degrade shearer performance and the reliability of key components. In particular, the cutting unit housing is a large welded structure with geometric discontinuities and is susceptible to deformation and fatigue damage. Therefore, investigating drum vibration and housing fatigue under multi-factor coupling is essential for structural optimization and operational reliability [4,5].

In recent years, researchers have made substantial progress in vibration-based fault diagnosis, structural optimization, cutting strategy optimization, and coal-rock identification. For example, Fan Hongwei et al [6] identified changes in the time-frequency domain characteristics of vibration signals when different types of faults occurred in the shearer’s cutting component’s transmission gears. Niu Naiping et al [7] applied a convolutional neural network model to diagnose faults in the shearer’s rocker arm gear based on time-domain vibration signals. However, due to the low accuracy and efficiency of fault diagnosis using raw time-domain vibration signals, scholars have proposed algorithms such as deep autoencoders [8] and improved empirical mode decomposition to denoise [9] vibration signals, significantly improving diagnostic accuracy. Additionally, some researchers have transformed vibration signals into images through time-frequency conversion algorithms, using them as inputs for deep learning models to extract deeper features for fault diagnosis [10–13].

Further studies have focused on the vibration characteristics of spiral drums and rocker arms. Xin Hongbao et al. [14] analyzed the modal characteristics of end plates with different pick arrangements and found that a hybrid arrangement reduced drum vibration. Zhang Yimin et al. [15] investigated the effects of five design variables, including rocker-arm wall thickness, on natural frequency through experimental modal analysis. Wang Yadong et al. [5] used time-frequency images of drum vibration acceleration to identify coal-rock cutting states and develop an adaptive control strategy. Zhao Lijuan et al. [4] combined drum and rocker-arm vibration signals with fuzzy control for coal-rock state identification and coordinated control of traction and drum speeds. Wang Haijian et al. [16] fused five signals to identify coal-rock interfaces, and Liu Jinshuo et al. [17] proposed lithology identification based on EEMD and a DE-optimized probabilistic neural network. These studies demonstrate the diagnostic value of vibration signals, but they do not quantify the coupled contributions of coal-rock properties, drum geometry, and operating parameters while linking the validated load source to housing fatigue.

Furthermore, the vibration of the coal-rock cutting process is transmitted through the spiral drum and transmission system to the shell, directly impacting its lifespan. Therefore, investigating the fatigue life of the cutting component shell under multi-factor coupling is crucial for optimizing shearer structure design and improving efficiency and safety. Li Xiaopeng [18] focused on the issue of planetary gear system failure caused by shearer cutting loads, conducting dynamic reliability research based on the stress-strength interference theory to provide theoretical support for the design and maintenance of shearer planetary gear systems. Zhao Lijuan [19] and others applied an improved PSO-BP algorithm for fatigue life analysis and prediction of the planetary frame in the shearer’s cutting component. Tian et al [20] developed a flexible-rigid coupled dynamic model for the shearer and performed dynamic simulations to acquire stress data for the rocker arm shell, planetary frame, and planetary shafts, using orthogonal experiments and PSO-BP neural networks to analyze critical reliability factors. These studies provide practical guidance for the reliability analysis and optimization design of key shearer components.

Researchers primarily investigate the vibration characteristics of shearer spiral drums and the service life of the cutting head housing through numerical simulations [21] and experiments [22,23]. Experimental methods are costly, time-consuming, and prone to data distortion. In contrast, numerical simulations can replicate extreme operating conditions, reveal the dynamic interactions between the drum and coal-rock, and capture deformation in weak areas of the housing, thereby accelerating the research process. However, limited model accuracy and reliability remain critical challenges for broader application.

The main innovative elements of this study are shown in Table 1.

**Table 1:** Comparison with previous studies and research gap addressed in this study.

Research focus	Representative studies	Main limitation	Present study
Vibration signals and fault diagnosis	[6–13]	Mainly identifies faults or coal-rock states from signals; load generation and housing fatigue are not jointly modeled.	Links coal-rock cutting loads, drum vibration, and housing fatigue in one DEM-MFBD workflow.
Drum or rocker-arm dynamics	[14–17,21–23]	Often considers modal or single-factor vibration behavior; multi-factor contribution and mechanism are insufficient.	Uses a five-factor mixed orthogonal design to quantify vibration sensitivity and physical mechanisms.
Reliability and fatigue of shearer components	[18–20,24]	Fatigue evaluation is usually separated from the validated cutting-load source.	Uses the validated coupled load spectrum for stress reliability and fatigue life prediction of the housing.
DEM contact calibration and cutting simulation	[25–33]	DEM parameters and cutting behavior are often discussed separately from fatigue design.	Combines calibrated coal-rock DEM, bidirectional EDEM-RecurDyn coupling, experimental validation, and fatigue analysis.

The comparison indicates that previous studies have provided useful foundations for fault diagnosis, structural dynamics, and fatigue analysis, but few have integrated coal seam heterogeneity, drum structure, motion parameters, vibration validation, and housing fatigue prediction within a unified framework.

To address this gap, this study focuses on the spiral drum and cutting housing of the MG2 × 55/250-BW thin coal seam shearer. The objectives are to: (1) construct a bidirectional DEM-MFBD rigid-flexible coupled model for coal seams containing gangue; (2) validate the simulated drum vibration response using cutting experiments; (3) quantify the contribution of coal seam, drum structure, and motion parameters to vibration through a mixed-level Taguchi orthogonal design and ANOVA; and (4) predict the fatigue weak zones and life of the cutting unit housing under the most severe working condition.

## 1. Construction of shearer cutting model for coal seam containing embedded coal and rock layers based on coupled dynamics

### 1.1 Determination of coal-rock parameters and coal wall model construction

Based on the coal seam occurrence conditions in a certain mining area, the mechanical properties of coal samples were tested according to the sampling principle and detection standards [3], and the physical and mechanical performance parameters of coal and rock samples were obtained Table 2.

**Table 2:** Physical and mechanical parameters of coal-rock.

Materials	Elasticity Modulus(MPa)	Density(kg/m³)	Poisson's Ratioμ	Uniaxial Compressive Strength(MPa)	Firmness Coefficient *f*
Coal	2010	1280	0.28	12	1.4
Aluminum Coal Rock (Intercalated Gangue 1)	3620	2460	0.24	30	3.5
Grey Coal Rock (Intercalated Gangue 2)	12100	2630	0.23	42	5.1
Limestone (With Gangue 3)	18300	2610	0.21	52	6.8
Roof	21500	2600	0.19	71	8.4

The discrete element model of coal seams should accurately represent the coal seam structure and mechanical properties. EDEM software, based on the Discrete Element Method (DEM), effectively simulates the interactions between granular materials and bonded substances, and has been widely used in discrete element analysis [25,26]. The Hertz-Mindlin with bonding model in EDEM generates cohesive forces by setting particle parameters, simulating the fracture and failure of coal and rock materials [27,28], and calculates normal and tangential contact forces between particles using the Hertz and Mindlin theories.

According to Hertz contact theory and the Hertz-Mindlin contact model used in DEM simulations [25–29], the relationship between particle interaction forces and displacement is derived as shown in equations (1) and (2):


$$\begin{array}{c} F=\frac{\text{4}}{\text{3}}E^{*}{\left(R^{*}\right)}^{1/2}\varepsilon^{3/2} \end{array}$$ (1)

$$\begin{array}{c} U={\left(\frac{3FR^{*}}{4E^{*}}\right)}^{1/3} \end{array}$$ (2)

The normal stiffness and tangential stiffness, as well as the normal and tangential contact forces between particles, can be calculated using equations (3) to (6) based on the Hertz-Mindlin no-slip contact formulation [29,30].


$$\begin{array}{c} k_{n}=\frac{2E}{3\left(1-\mu^{\text{2}}\right)}{\left(R^{*}\right)}^{1/2} \end{array}$$ (3)

$$\begin{array}{c} k_{s}={\left(\frac{E}{1+\mu }\right)}^{2/3}\frac{{\left(12\left(1-\mu \right)R^{*}F_{n}\right)}^{1/3}}{2-\mu } \end{array}$$ (4)

$$\begin{array}{c} F_{n}=k_{n}{U_{n}}^{3/2} \end{array}$$ (5)

$$\begin{array}{c} F_{s}=k_{s}{U_{s}}^{3/2} \end{array}$$ (6)

Where: F represents the force between particles (N); $E^{*}$is the equivalent elastic modulus between particles (MPa); $R^{*}$is the contact radius of particles (mm); εis the overlap between particles; $U_{n}$and $U_{s}$represent normal and tangential displacements (mm); Eis the elastic modulus of the particles (MPa); μis the Poisson's ratio of the particles.

**Fig 1:**
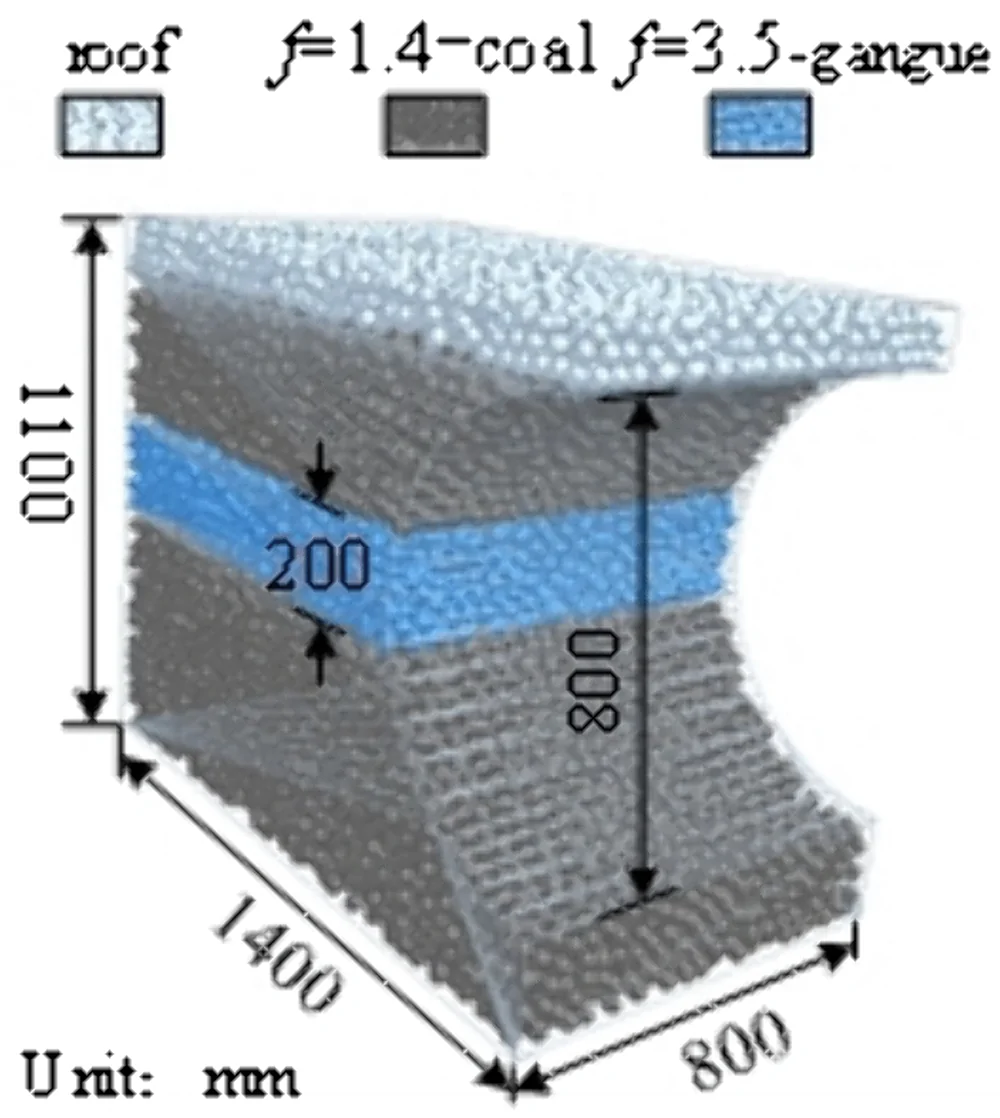
Discrete element model of coal wall.

The contact and cohesive parameters for particles can be calibrated based on prior research results [31], using simulations of angle of repose, uniaxial compression, and physical tests, and are not discussed further here. In EDEM, a discrete element model for coal seams with intercalated gangue is established by importing a particle factory model, defining material properties for coal and rock particles, and setting contact and cohesive parameters, as shown in Fig 1.

### 1.2 Construction of DEM-MFBD bidirectional coupling model for shearer cutting

**Fig 2:**
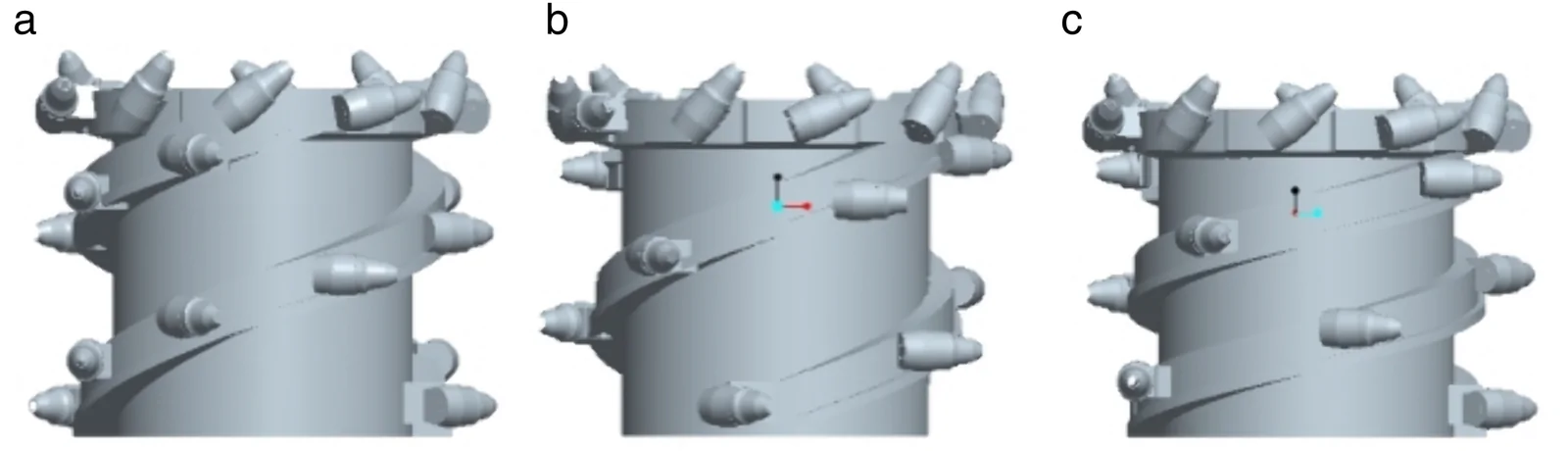
Model diagrams of spiral drums with different structures. (a) 13° ascending-angle sequential type (b) 13° cross type (c) 11° cross type.

Using the MG2 × 55/250-BW shearer as the prototype, drum models with different spiral angles and pick arrangements were developed in Pro/E (Fig 2). The 13° sequential drum is the actual prototype and therefore serves as the reference configuration. Based on the preliminary research findings of the project team [32] uses 11°, 13°, and 15° to form a symmetric engineering range around the 13° prototype and to separate the local blade-angle effect from pick arrangement and motion parameters. Table 3 distinguishes these structural comparison models from the orthogonal-design levels.

**Table 3:** Main parameters of drum.

Drum Parameter	Value	Unit	Drum Parameter	Value	Unit
Drum Diameter	800	mm	Hub Outer Diameter	465	mm
Spiral Blade Height	68	mm	Hub Inner Diameter	425	mm
Spiral Blade Thickness	90	mm	Cutter Tooth Arrangement	Sequential/Cross	—
Spiral Blade Angle	11/13/15	°	Drum Width	630	mm
Number Of Blades	2	—	Teeth Per Line	1 or 2	—
Cutter Tooth Length	80	mm	Blade Rotation Direction	Right	—
End Cap Thickness	25	mm	Cutter Tooth Installation Angle	45	°
End Cap Diameter	475	mm	Total Number Of Cutter Teeth	24/22	—
Outer Diameter Of The End Plat	625	mm	The Axial Inclination Angle Of The Second Cutting Line Tooth Of The End Plate	3	°
The Axial Inclination Angle Of The First Cutting Tooth Of The End Plate	0	°	The Axial Inclination Angle Of The Cutting Teeth On The Fourth Cutting Line Of The End Plate	12	°
The Axial Inclination Angle Of The Cutting Teeth On The Third Cutting Line Of The End Plate	8	°	The Axial Inclination Angle Of The Fifth Cutting Line And Cutting Teeth Of The End Plate	15	°

**Fig 3:**
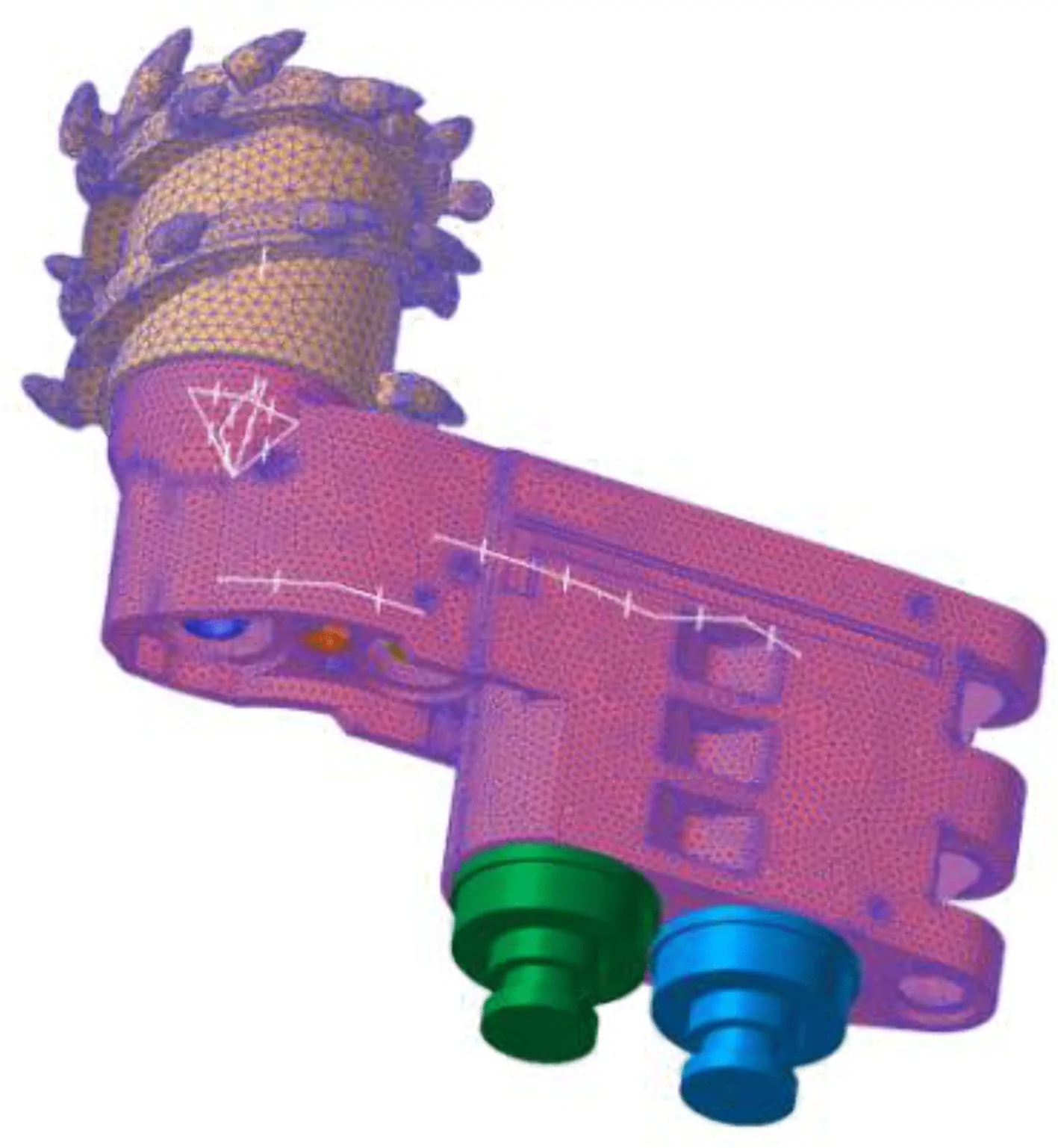
The rigid-flexible coupling simulation model of shearer.

During the cutting process, the spiral drum and cutting shell directly interact with the coal wall containing embedded gangue, experiencing high-impact, nonlinear loading. Stress concentration and deformation cannot be ignored. In RecurDyn, the Mesher module was used for flexible processing. Based on reference [33], contact parameters such as stiffness, damping, maximum penetration, and nonlinear indices were set, resulting in the final rigid-flexible coupling simulation model of the shearer, shown in Fig 3.

For the flexible cutting housing, mesh independence was considered during model development. The mesh was refined until the changes in maximum equivalent stress and dominant vibration response were within the engineering tolerance used for the coupled simulation. To control computational cost, the housing was modeled as a flexible body while non-critical transmission components were retained as rigid bodies; the same contact, time-step, and boundary-condition settings were then used for all orthogonal cases. This treatment reduces numerical uncertainty from mesh density and ensures that the response differences in the orthogonal tests mainly reflect factor-level changes rather than discretization effects.

Sensitivity to the five physical and operating factors is quantified in Section 2.2 through the Taguchi main effects and ANOVA. Numerical uncertainty also arises from DEM contact calibration, material scatter, time-step selection, the rigid-flexible idealization, and mesh density.

**Fig 4:**
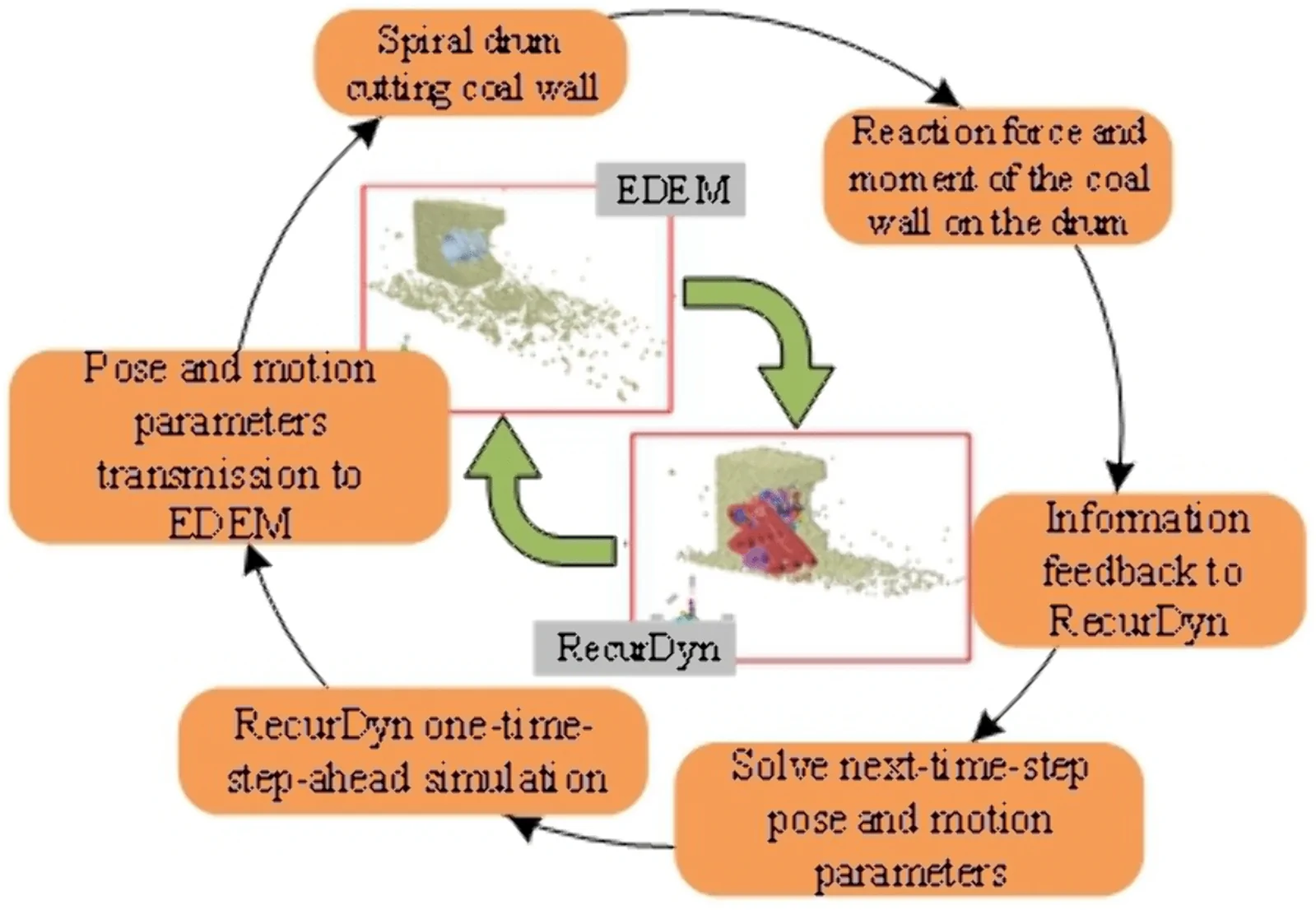
EDEM-RecurDyn coupling calculation principle.

The coupling interface between EDEM and RecurDyn enables the seamless data exchange between the discrete element model of the coal wall and the rigid-flexible coupling simulation model of the shearer, as illustrated in Fig 4. As shown in Fig 4, the simulation employs a time-stepping forward mechanism: RecurDyn first completes a time step simulation, transferring the shearer’s position and motion parameters to EDEM, which then drives the cutting of the discrete coal wall. Based on particle contact theory, EDEM calculates the reaction forces and moments exerted by the coal wall on the drum, which are fed back to RecurDyn. RecurDyn, in turn, combines the feedback loads with its own driving force to solve for the next time step’s position and motion parameters, which are subsequently sent back to EDEM, thereby achieving the bidirectional coupling of dynamic coal cutting.

### 1.3 Coupled simulation analysis and feasibility verification

**Fig 5:**
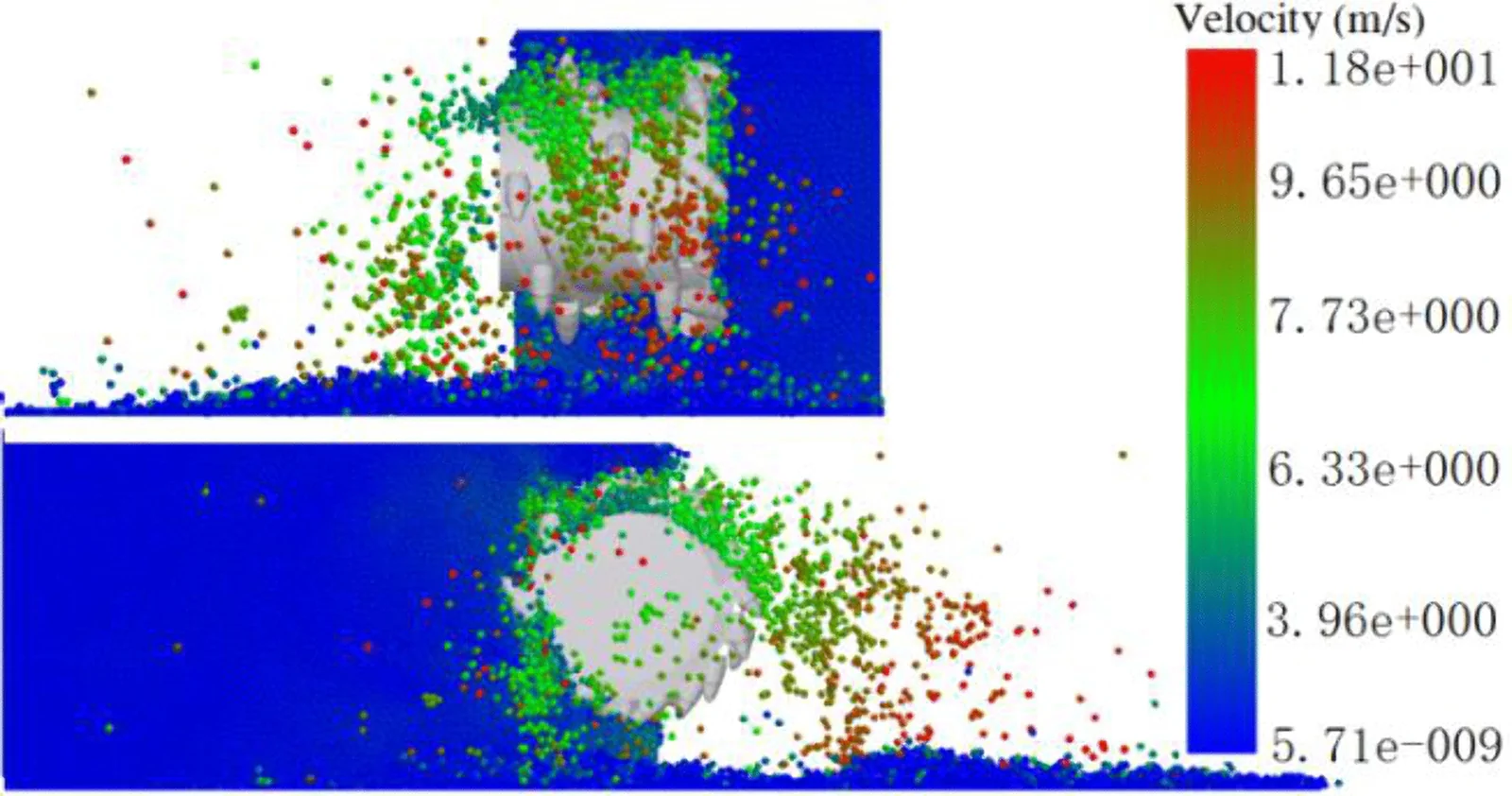
Simulation cutting effect. (a) Vibration characteristics based on simulation (b) Vibration characteristics based on the test.

**Fig 6:**
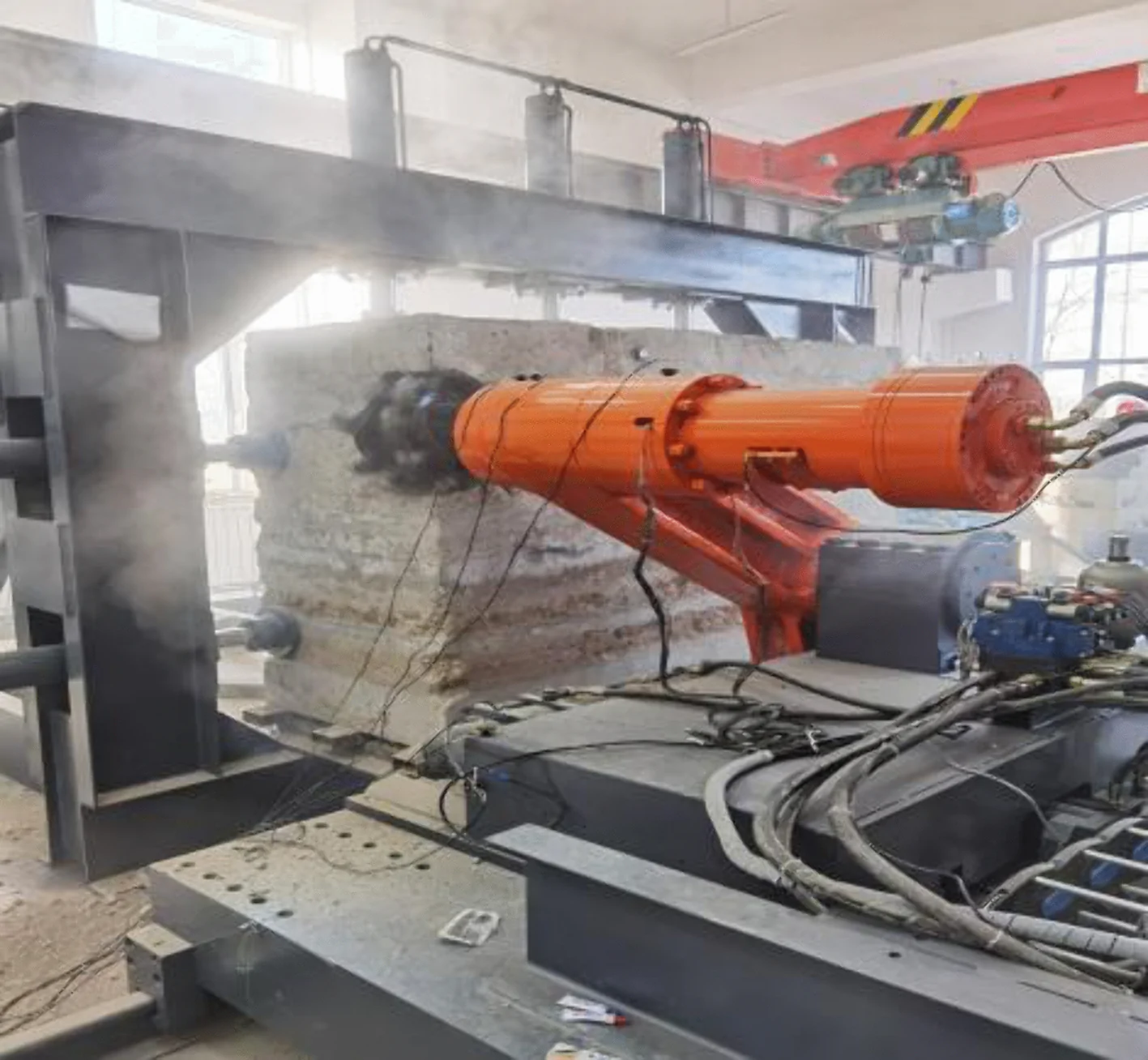
Experimental cutting process. (a) Vibration characteristics based on simulation (b) Vibration characteristics based on the test.

**Fig 7:**
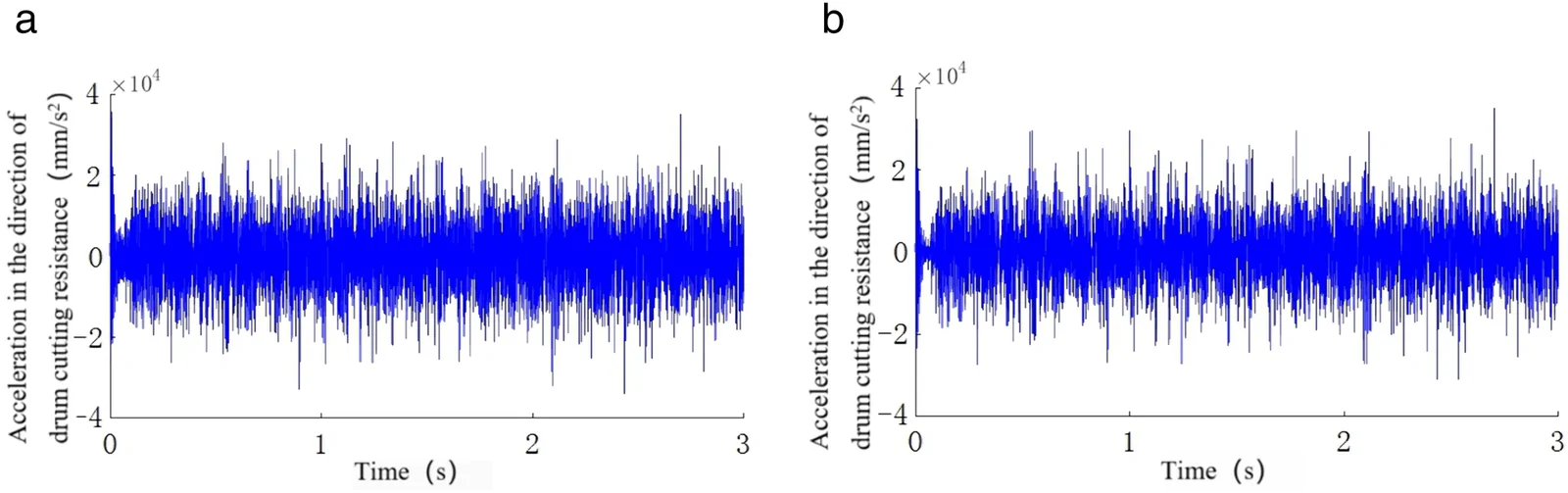
Drum vibration characteristics based on simulation and experiment.

Using similarity theory, an experimental coal wall model with properties consistent with the EDEM model in Fig 2 was constructed. A shearer with a 13 deg spiral blade angle was used for simulation, with a traction speed of 4 m/min and a spiral drum speed of 90 r/min. The EDEM-RecurDyn simulation cutting effect is shown in Fig 5, and the experimental cutting process is shown in Fig 6. The drum vibration response was selected as the validation indicator [33]. The time-domain signals are shown in Fig 7, and the statistical vibration characteristics are presented in Table 4.

**Table 4:** Drum vibration characteristic values.

Feature Parameter	Simulation Value (mm/s^2^)	Experimental Value (mm/s^2^)	Relative Error (%)
RMS Value	6421.56	6167.91	3.95
Maximum Value	35541.27	34051.94	4.19
Minimum Value	−32785.29	−31828.52	2.92

In Fig 5, the high-velocity particles are mainly concentrated around the pick-coal and pick-gangue contact regions. The discontinuous particle ejection and the asymmetric velocity field indicate that the drum experiences intermittent impact loads when cutting through the gangue layer. This behavior is consistent with the experimental cutting process in Fig 6, where the gangue layer causes local fragmentation resistance and transient load fluctuation. Therefore, the simulation reproduces not only the macroscopic cutting trajectory but also the dynamic particle-ejection pattern that drives drum vibration.

A comparative analysis of the vibration characteristics in Fig 7 and the feature values in Table 4 reveals that the vibration waveform of the drum is highly consistent between simulation and experiment. The simulation value is slightly higher in amplitude, with the maximum relative error of the vibration characteristic values being only 4.19%, which is within an acceptable range. This confirms the feasibility and reliability of the EDEM-RecurDyn bidirectional coupled simulation for vibration prediction.

## 2. Study on the vibration characteristics of the coal mining machine spiral drum

### 2.1 Simulation and working condition design

The reasonable ranges of each factor and corresponding levels are determined based on the preliminary research findings [34]. Five factors were selected to represent the principal sources and transmission paths of cutting vibration: gangue hardness coefficient A describes coal-rock heterogeneity; blade spiral angle B and pick arrangement E describe drum structure; and traction speed C and drum speed D describe operating conditions. The A levels (3.5, 5.1, and 6.8) correspond to the three measured gangue materials in Table 2. The B levels (11°, 13°, and 15°) are centered on the 13° prototype and remain within the available drum geometry. The C levels (3.5, 4.0, and 4.5 m/min) and D levels (75, 90, and 105 r/min) bracket the nominal test condition of 4.0 m/min and 90 r/min without exceeding the prototype operating range. Factor E contains the two realizable pick layouts, sequential and cross-type. The factor levels are summarized in Table 5.

**Table 5:** Factor levels.

Level	Gangue Hardness Coefficient /A	∘Spiral Blade Angle / B (°)	Tractio Speed /C(m/min)	Spiral Drum Speed /D(r/min)	Cutter Arrangement /E
1	3.5	11	3.5	75	Sequential
2	5.1	13	4.0	90	Crossed
3	6.8	15	4.5	105	—

A standard Taguchi L18(3^4^ × 2^1^) orthogonal array was used to generate 18 combinations for four three-level factors (A-D) and one two-level factor (E). The matrix and fixed-effects main-effect ANOVA were independently recalculated in Python 3.12 using NumPy 2.3.5. An EDEM-RecurDyn coupled simulation was performed for each combination. The maximum, minimum, and Root Mean Square (RMS) vibration accelerations in the cutting-resistance direction were extracted. RMS represents the effective vibration energy over the complete cutting interval and is less sensitive to a single transient than the maximum value. The sums K, means k, ranges R, and factor-level variances S were calculated for descriptive screening; ANOVA was then used for inferential comparison. Tables 6–8 present the design, responses, main effects, and ANOVA results.

**Table 6:** Experimental design and orthogonal test results.

Test plan	Factor coding					MaxValue /(mm/s^2^)	Min Value /(mm/s^2^)	RMS Value /(mm/s^2^)
	*A*	*B*	*C*	*D*	*E*			
1	1	1	1	1	1	33098.76	−30501.65	5980.25
2	1	2	2	2	1	35541.27	−32785.29	6421.56
3	1	3	3	3	1	38701.43	−35981.62	6992.53
4	2	1	1	2	1	39954.90	−37154.76	7219.01
5	2	2	2	3	1	42356.76	−39601.34	7652.98
6	2	3	3	1	1	43156.87	−40321.75	7797.54
7	3	1	2	1	1	47213.79	−44358.21	8530.54
8	3	2	3	2	1	49526.97	−46701.65	8948.48
9	3	3	1	3	1	46795.09	−44087.54	8454.89
10	1	1	3	3	2	35763.54	−32990.08	6461.72
11	1	2	1	1	2	29786.04	−27003.07	5381.71
12	1	3	2	2	2	32905.75	−30123.89	5945.38
13	2	1	2	3	2	39598.91	−36872.65	7154.69
14	2	2	3	1	2	39754.82	−36993.05	7182.86
15	2	3	1	2	2	36912.43	−34175.58	6669.30
16	3	1	3	2	2	46992.98	−44187.91	8490.64
17	3	2	1	3	2	43312.64	−40754.52	7825.68
18	3	3	2	1	2	44108.43	−41408.76	7969.47

**Table 7:** Factor influence analysis.

	Max Value/(×10^4^mm/s^2^)					Min Value/(×10^4^mm/s^2^)					RMS Value/(×10^4^mm/s^2^)				
	*A*	*B*	*C*	*D*	*E*	*A*	*B*	*C*	*D*	*E*	*A*	*B*	*C*	*D*	*E*
*K*_1_	20.58	24.26	22.99	23.71	37.63	−18.94	−22.61	−21.37	−22.06	−35.15	3.72	4.38	4.15	4.28	6.80
*K*_2_	24.17	24.03	24.17	24.18	34.91	−22.51	−22.38	−22.52	−22.51	−32.45	4.37	4.34	4.37	4.37	6.31
*K*_3_	27.79	24.26	25.39	24.65	–	−26.15	−22.61	−23.72	−23.03	–	5.02	4.38	4.59	4.45	–
*k*_1_	3.43	4.04	3.83	3.95	4.18	−3.16	−3.77	−3.56	−3.68	−3.91	0.62	0.73	0.69	0.71	0.76
*k*_2_	4.03	4.00	4.03	4.03	3.88	−3.75	−3.73	−3.75	−3.75	−3.61	0.73	0.72	0.73	0.73	0.70
*k*_3_	4.63	4.04	4.23	4.11	–	−4.36	−3.77	−3.95	−3.84	–	0.84	0.73	0.76	0.74	–
*R*	1.20	0.039	0.40	0.16	0.30	1.20	0.037	0.39	0.16	0.30	0.22	0.0071	0.072	0.028	0.055
*S*	0.60	0.022	0.20	0.0784	0.21	0.60	0.0216	0.19	0.0808	0.21	0.11	0.0040	0.0362	0.0142	0.0386

**Table 8:** ANOVA results and contribution rates for vibration responses.

Response	Factor	df	Contribution (%)	F value	p value
Maximum	*A*	2	81.68	60438.11	<0.001
Maximum	*B*	2	0.11	83.55	<0.001
Maximum	*C*	2	9.07	6707.70	<0.001
Maximum	*D*	2	1.39	1027.89	<0.001
Maximum	*E*	1	7.74	11460.51	<0.001
Minimum	*A*	2	82.03	65287.39	<0.001
Minimum	*B*	2	0.11	84.25	<0.001
Minimum	*C*	2	8.71	6933.74	<0.001
Minimum	*D*	2	1.49	1183.12	<0.001
Minimum	*E*	1	7.66	12188.54	<0.001
RMS	*A*	2	81.68	60427.39	<0.001
RMS	*B*	2	0.11	83.53	<0.001
RMS	*C*	2	9.07	6706.49	<0.001
RMS	*D*	2	1.39	1027.70	<0.001
RMS	*E*	1	7.74	11458.48	<0.001

### 2.2 Analysis of simulation results

#### 2.2.1 Factor influence degree and ANOVA.

The range *R* measures the change in the mean vibration response across the levels of each factor. For the maximum, minimum, and RMS vibration accelerations in Table 7, the range ranking is *R*_*A*_ *> R*_*C*_ *> R*_*E*_ *> R*_*D*_ *> R*_*B*_*.* Thus, gangue hardness is the dominant factor, traction speed and cutter arrangement are secondary factors, drum speed has a smaller effect, and spiral blade angle has the least effect within the selected range.

The factor-level variance *S* gives the ranking *S*_*A*_ *> S*_*E*_ *> S*_*C*_ *> S*_*D*_ *> S*_*B*_*.* This metric also identifies gangue hardness as the dominant factor and blade angle as the weakest factor, while cutter arrangement and traction speed have similar secondary effects. Their order differs slightly because *E* has two levels and *C* has three levels; the subsequent ANOVA accounts for their different degrees of freedom.

Overall, the range and factor-level variance analyses consistently identify gangue hardness as the dominant excitation source and blade spiral angle as the least influential factor. ANOVA is used below to quantify the contribution and statistical significance of each factor Table 8.

The fixed-effects ANOVA confirms the range-analysis ranking. For the maximum response, R^2^ = 0.999946 and adjusted R^2^ = 0.999885; for the minimum response, R^2^ = 0.999950 and adjusted R^2^ = 0.999893; and for RMS, R^2^ = 0.999946 and adjusted R^2^ = 0.999885. Gangue hardness contributes 81.68%, 82.03%, and 81.68% to the maximum, minimum, and RMS responses, respectively. Traction speed contributes 9.07%, 8.71%, and 9.07%; pick arrangement contributes 7.74%, 7.66%, and 7.74%; drum speed contributes 1.39%, 1.49%, and 1.39%; and blade angle contributes approximately 0.11% to all three responses.

#### 2.2.2 Factor influence trend and physical interpretation.

**Fig 8:**
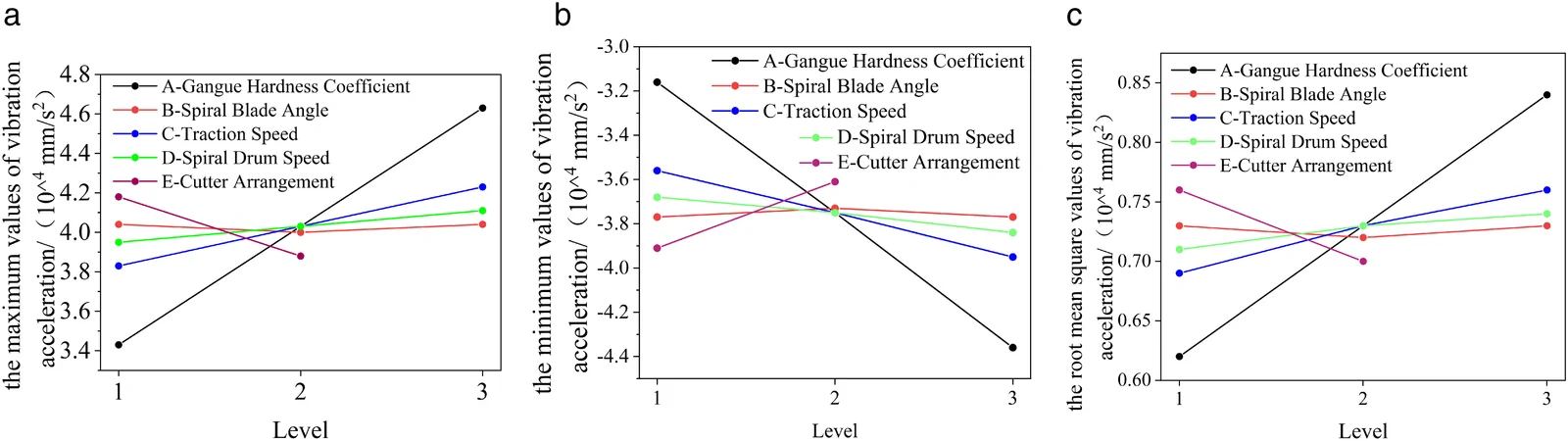
Factor trend analysis. (a)The influence of factors on the maximum value of vibration acceleration; (b)The influence of factors on the minimum value of vibration acceleration; (c)The influence of factors on the root mean square value of vibration acceleration.

By plotting the levels of the gangue hardness coefficient, blade spiral angle, cutter arrangement, traction speed, and spiral drum speed on the x-axis, and the maximum, minimum, and RMS values of the vibration acceleration on the y-axis, the trend of each factor's influence on the vibration characteristics of the spiral drum is shown in Fig 8.

From Fig 8(a), the maximum vibration acceleration increases almost linearly as the gangue hardness coefficient increases, with the largest increase of 34.99%. Physically, harder gangue increases contact stiffness and fragmentation resistance, which strengthens the transient impact force acting on the picks. Traction speed also increases the maximum acceleration by 10.44% because a higher feed rate increases the instantaneous cutting thickness and engagement load. Drum speed has a smaller effect (4.05%) because the increased contact frequency is partly offset by reduced cutting depth per pick. The spiral blade angle produces only a slight non-monotonic change within the selected range, indicating that the load magnitude is more sensitive to coal-rock hardness and pick engagement than to blade angle. The maximum acceleration of the sequential pick arrangement is about 4.18 × 10^4^ mm/s^2^, whereas that of the cross arrangement is about 3.88 × 10^4^ mm/s^2^, indicating a 7.73% reduction due to phase dispersion of pick impacts.

As shown in Fig 8(b), the minimum vibration acceleration becomes more negative as the gangue hardness coefficient increases, with a maximum change of 37.97%. The negative value does not represent a negative vibration intensity; rather, it indicates acceleration in the direction opposite to the defined positive cutting-resistance axis. This reverse peak is caused by rebound, intermittent pick disengagement, and inertia after the drum strikes the harder gangue. Therefore, the magnitude of the negative peak is a useful indicator of bidirectional impact severity. Compared with the sequential arrangement, the cross-type arrangement increases the minimum value from about −3.91 × 10^4^ mm/s^2^ to −3.61 × 10^4^ mm/s^2^, reducing the reverse impact by 8.31%.

As shown in Fig 8(c), the RMS vibration acceleration increases by 35.48% as the gangue hardness coefficient increases, confirming that harder gangue raises the effective vibration energy during the whole cutting process. Traction speed and drum speed increase the RMS response by 10.14% and 4.23%, respectively, while the blade spiral angle remains a secondary factor. The RMS value of the sequential pick arrangement is approximately 0.76 × 10^4^ mm/s^2^, whereas that of the cross arrangement is about 0.70 × 10^4^ mm/s^2^, corresponding to an 8.57% reduction. This demonstrates that the cross arrangement can smooth the load distribution and reduce cumulative vibration energy.

## 3. Fatigue life prediction of cutting section housing

The cutting process of the shearer’s cutting drum housing is subjected to intense impact and vibration, resulting from the combined effects of multiple factors. This leads to fatigue damage, as the cyclic loads causing fatigue failure are often significantly lower than the maximum loads experienced by the housing. Therefore, fatigue analysis of the component should be conducted using stress-based fatigue theory.

### 3.1 Reliability analysis of the cutting section housing

To better visualize the reliability of the cutting section housing, a functional relationship between the maximum equivalent stress and the reliability is established. The Gaussian-type membership function is used to map the maximum equivalent stress values obtained from the simulation under different working conditions to the reliability values, as shown in equation (7):


$$\begin{array}{c} g\left(x,c,\sigma \right)=exp\left[-\frac{\text{1}}{\text{2}}{\left(\frac{x-c}{\sigma }\right)}^{\text{2}}\right] \end{array}$$ (7)

Where:g(x,c,σ)is the membership function of variable *x*,*C* is the mean value,σ is the standard deviation.

A Gaussian membership function is used because the stress-reliability indicator should decrease smoothly as maximum equivalent stress exceeds the allowable stress.Compared with triangular or trapezoidal membership functions, the Gaussian function avoids abrupt changes at artificial thresholds and is more suitable for representing continuous uncertainty caused by material scatter, dynamic load fluctuation, and model simplification.

The domain of the Gaussian membership function is defined as 3σ, σ and the function is given by:


$$\begin{array}{c} \sigma =\frac{C_{max}-C_{min}}{\text{3}} \end{array}$$ (8)

Where:$C_{\text{max}}$and$C_{\text{min}}$represent the maximum and minimum values of xwithin the domain.

The shearer cutting section housing is made from ZG20SiMn material, with a yield strength of 322.8 MPa [30]. The safety factor is generally taken to be between 1.5 and 2.5, with a value of 2.5 being used here. This gives the allowable stress of 129.12 MPa. Based on this, the stress-reliability membership function for the housing is constructed, as shown in equation (9):


$$\begin{array}{c} R_{s}=\mu_{s}\left(x\right)=\left\{ \begin{array}{c} @l@1\begin{array}{cccc} & & & \begin{array}{ccc} & & \begin{array}{cccc} & & & 0<x_{\sigma }\leq 129.12\text{MPa} \end{array} \end{array} \end{array}\\ exp\left[-\frac{\text{1}}{\text{2}}{\left(\frac{x_{\sigma }-129.12}{64.56}\right)}^{\text{2}}\right]\begin{array}{cc} & 129.12\text{MPa}<x_{\sigma }<322.8\text{MPa} \end{array} \end{array}\right. \end{array}$$ (9)

Where:$x_{\sigma }$ is the maximum stress applied to the cutting section housing.

**Fig 9:**
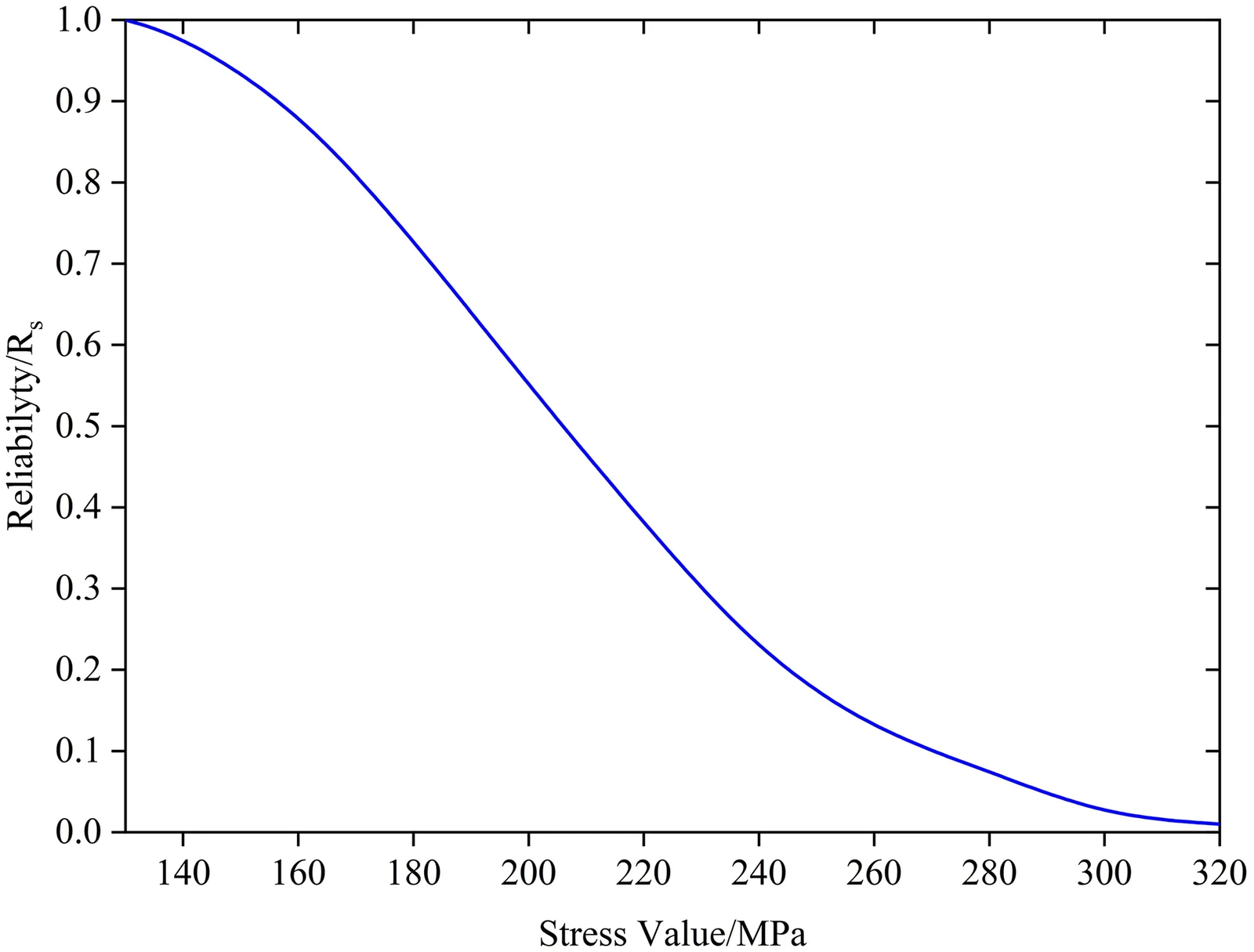
Reliability function curve of cutting section housing.

Using equation (9), the reliability function $R_{\text{s}}$ of the cutting section housing is calculated, and the resulting reliability curve is shown in Fig 9.

By extracting the stress values from the cutting section housing, the corresponding reliability values for each working condition are obtained using equation (9). The results for the 18 orthogonal test schemes are shown in Table 9.

**Table 9:** Maximum equivalent stress and reliability data for shell orthogonal test.

Test Scheme	Factor Code					Max Equivalent Stress (MPa)	Reliability
	*A*	*B*	*C*	*D*	*E*		
1	1	1	1	1	1	62.16	1
2	1	2	2	2	1	68.62	1
3	1	3	3	3	1	81.18	1
4	2	1	1	2	1	92.17	1
5	2	2	2	3	1	100.83	1
6	2	3	3	1	1	106.56	1
7	3	1	2	1	1	140.80	0.9838
8	3	2	3	2	1	154.73	0.9243
9	3	3	1	3	1	128.95	1
10	1	1	3	3	2	70.86	1
11	1	2	1	1	2	53.23	1
12	1	3	2	2	2	60.25	1
13	2	1	2	3	2	85.64	1
14	2	2	3	1	2	88.80	1
15	2	3	1	2	2	75.16	1
16	3	1	3	2	2	134.77	0.9962
17	3	2	1	3	2	110.73	1
18	3	3	2	1	2	116.76	1

Based on the orthogonal test results in Table 9, it is determined that Scheme 8 corresponds to the most severe working condition, with a maximum equivalent stress of 154.73 MPa and a reliability of 0.9243. This working condition is used as the basis for fatigue life prediction of the cutting section housing.

### 3.2 Fatigue cumulative damage theory

The fatigue life of components under constant load can be estimated using the S-N curve [35]. However, the cutting drum housing of the shearer is subjected to variable-amplitude impact loads. To address fatigue under such loading conditions, the Palmgren-Miner linear damage accumulation theory, combined with the rainflow counting method, is employed. The total damage calculation formula for the studied component is expressed as Equation (10):


$$\begin{array}{c} D=\frac{m_{\text{1}}}{M_{\text{1}}}+\frac{m_{\text{2}}}{M_{\text{2}}}\cdots +\frac{m_{n}}{M_{n}}=\sum_{i=1}^{k}\frac{m_{i}}{M_{i}} \end{array}$$ (10)

Where:*D* is the total damage to the component,$m_{i}$is the number of cycles for load *i*,*N*_*i*_ is the fatigue life at load *i*,*k* is the number of load stress amplitudes.

The total damage is inversely related to the life cycle *M*, as given by equation (11):


$$\begin{array}{c} M=\frac{\text{1}}{D}=\frac{\text{1}}{\sum_{i=1}^{k}\frac{m_{i}}{M_{i}}} \end{array}$$ (11)

### 3.3 Fatigue life prediction

The multiaxial stress history was processed using the criterion labeled ‘stress-based Manson-Coffin’ in RecurDyn/Durability. The displayed Equation (12) has the Basquin-type elastic stress-life form, linking stress amplitude to reversals to failure It is distinct from the full Coffin-Manson strain-life equation because no plastic-strain term is included. Accordingly, the present calculation is an elastic stress-life estimate suitable for locating relative fatigue weak zones; low-cycle plasticity, corrosion-fatigue effects, weld defects, and crack growth require separate material data and validation.


$$\begin{array}{c} \frac{\Delta \sigma }{\text{2}}={\sigma^{\prime }}_{f}{\left(2M_{f}\right)}^{b} \end{array}$$ (12)

where Δσ/2 is the stress amplitude, σ′f is the fatigue-strength coefficient, 2Nf is the number of reversals to failure, and b is the fatigue-strength exponent.

**Fig 10:**
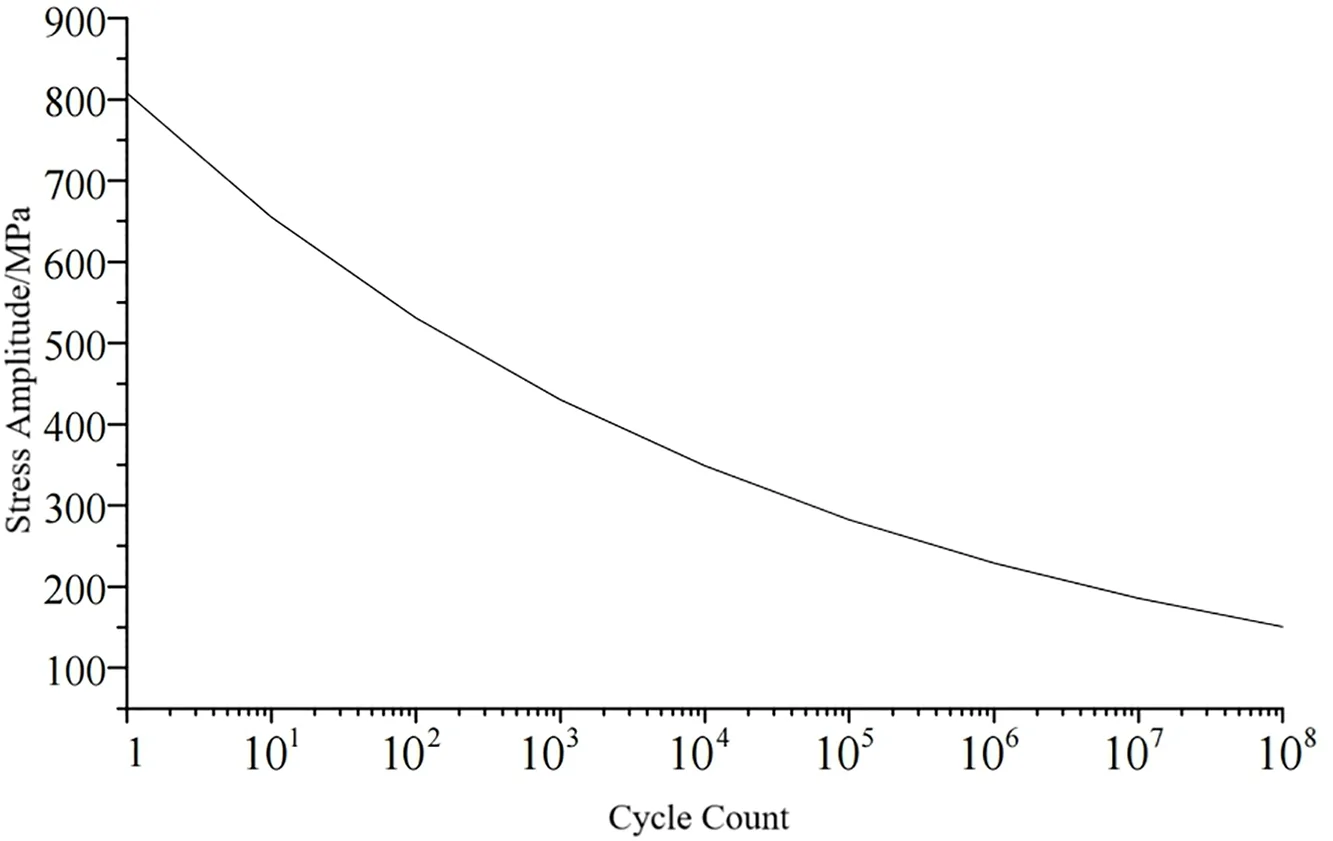
S-N curve of shearer cutting section housing.

For the ZG20SiMn material used in the shearer housing, the fatigue strength coefficient is determined to be 861 MPa, and the fatigue strength exponent is −0.0912. The S-N curve for the housing material is fitted as shown in Fig 10.

For the multiaxial stress history, Equation (13) defines the correlation coefficient γ between the two principal loading-direction stress sequences, following the fatigue post-processing formulation used in RecurDyn. Here, i denotes the time-step index and n is the number of samples.


$$\begin{array}{c} \gamma =\frac{\sum_{i}^{n}{\left(\sigma_{x}\cdot \sigma_{y}\right)}_{i}}{\sum_{i}^{n}{\left(\sigma_{x}\cdot \sigma_{x}\right)}_{i}} \end{array}$$ (13)

Where:*i* is each time step;*n* is the total number of time steps;*x* and *y* are the stresses in the primary and secondary loading directions.

Rainflow counting is applied to the principal loading-direction history in accordance with ASTM E1049. The equivalent stress amplitude and mean stress are then updated using Equations (14) and (15) according to the multiaxial correction implemented in RecurDyn. These transformations retain the sign and correlation of the two stress components before the Basquin curve and Palmgren-Miner accumulation are applied.

This fatigue workflow converts the validated dynamic load spectrum into stress amplitudes, counts the variable-amplitude cycles, and accumulates damage through the Miner rule. The approach is appropriate for identifying relative weak zones and comparing working conditions, but it assumes linear cumulative damage and does not explicitly include weld defects, residual stress, corrosion, or crack-growth behavior. These assumptions should be considered when applying the predicted life to field maintenance decisions [36].


$$\begin{array}{c} \frac{\Delta \overset{―}{\sigma }}{\text{2}}=\left(\sqrt{1-\gamma +\gamma^{\text{2}}}\right)\frac{\Delta \sigma }{\text{2}} \end{array}$$ (14)

$$\begin{array}{c} \overset{―}{\sigma_{m}}=\sigma_{m}\left(1+\gamma \right) \end{array}$$ (15)

### 3.4 Mesh independence verification

**Fig 11:**
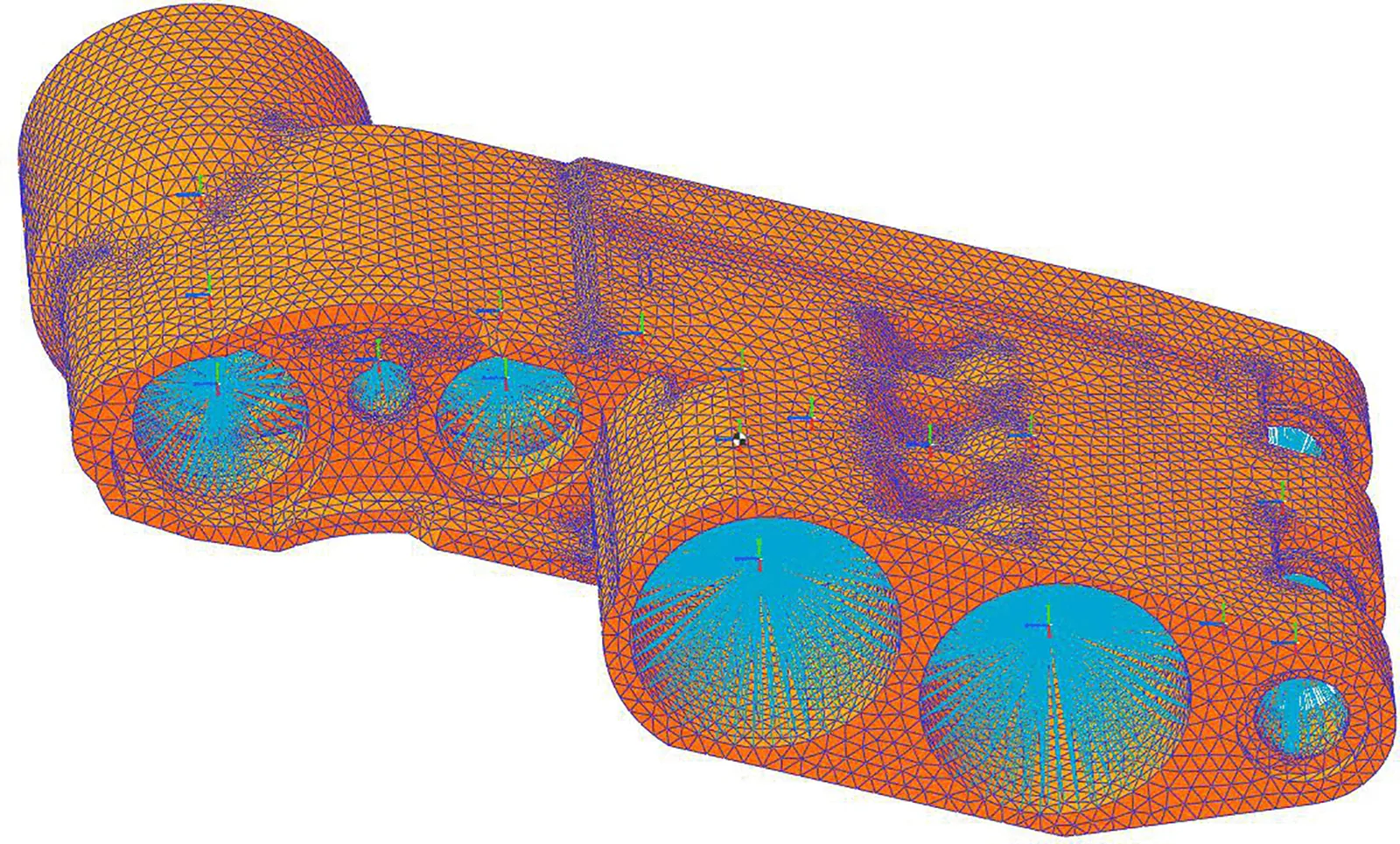
Mesh generation rendering.

A mesh independence study was carried out for the numerical simulation of shell structures, with five sets of graded shell mesh sizes (5–10 mm, 5–15 mm, 5–20 mm, 5–25 mm, 5–30 mm) established. Coarse meshes (5–25 mm and 5–30 mm) produced obvious calculation deviations due to insufficient discretization accuracy in regions with shell curvature and high stress gradients. After mesh refinement down to 5–20 mm and smaller sizes, the relative error of target physical quantities fell within an acceptable range; nevertheless, ultra-fine meshes drastically increased element count, computational resource consumption and simulation duration. Taking the dimension range of 5–20 mm as an example, the meshing scheme is illustrated in Fig 11.

**Fig 12:**
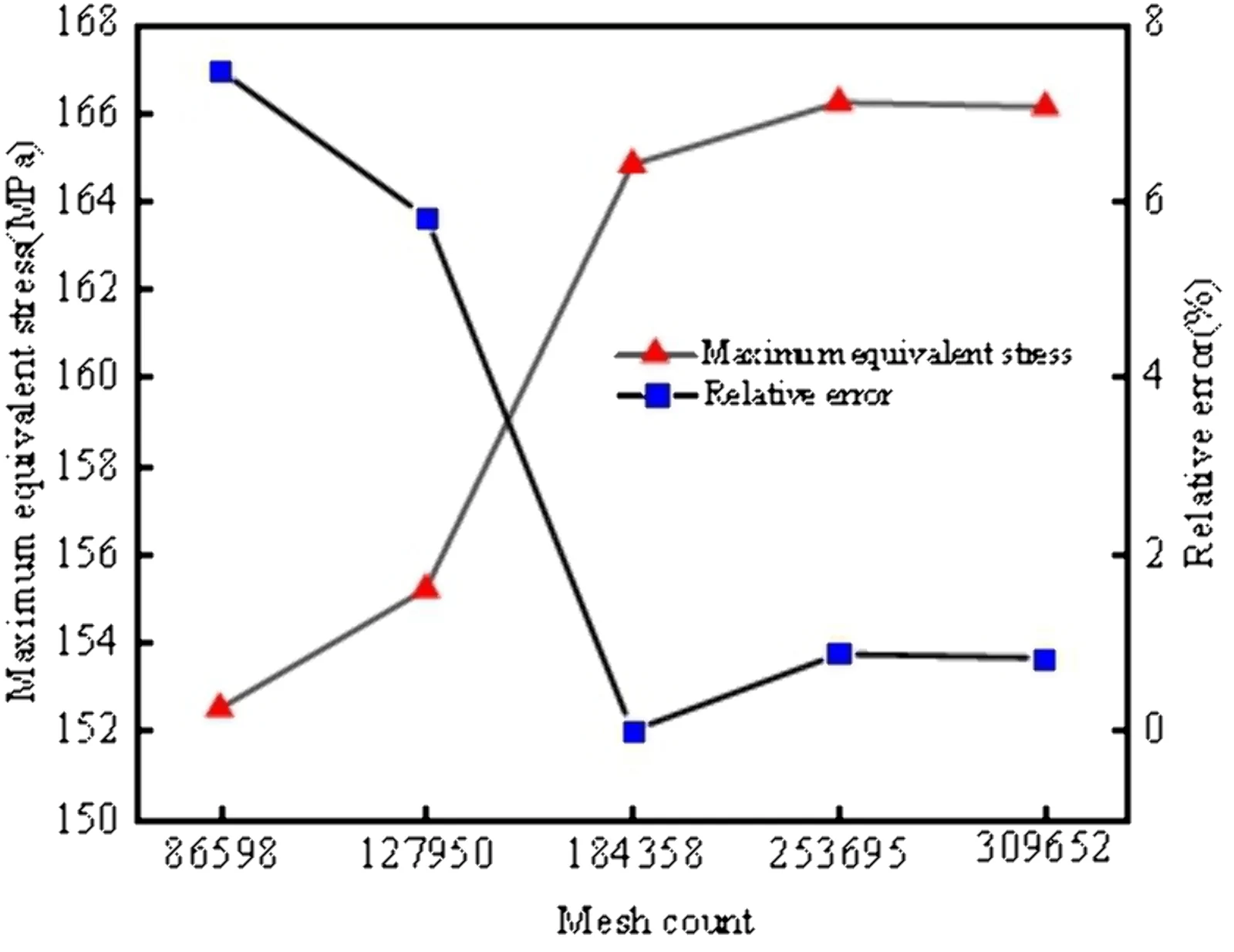
Grid independence verification analysis.

According to the verification data in Table 10, the maximum equivalent stress of the shell rose and gradually stabilized with the increase of mesh quantity, while the relative error of maximum equivalent stress declined and converged. The finest Mesh Model 4 and Model 5 only exhibited tiny relative errors of 0.87% and 0.82% compared with Model 3. Balancing simulation accuracy, computational cost and engineering practicability, the 5–20 mm mesh (Model 3) was selected as the baseline mesh scheme for subsequent numerical analysis. This mesh can accurately capture local stress concentration and deformation characteristics of the shell, achieving a favorable trade-off between precision and computational efficiency. The equivalent stress contour of the shell and mesh independence verification results are illustrated in Fig 11 and Fig 12, respectively.

**Table 10:** Shell grid independence verification.

Mesh Model	Model 1 (5–30 mm)	Model 2 (5–25 mm)	Model 3(5–20 mm)	Model 4 (5–15 mm)	Model 5 (5–10 mm)
Number of Mesh Elements	86598	127950	184358	253695	309652
Maximum Equivalent Stress / MPa	152.5	155.2	164.8	166.2	166.1
Relative Error / %	7.47	5.81	—	0.87	0.82

**Fig 13:**
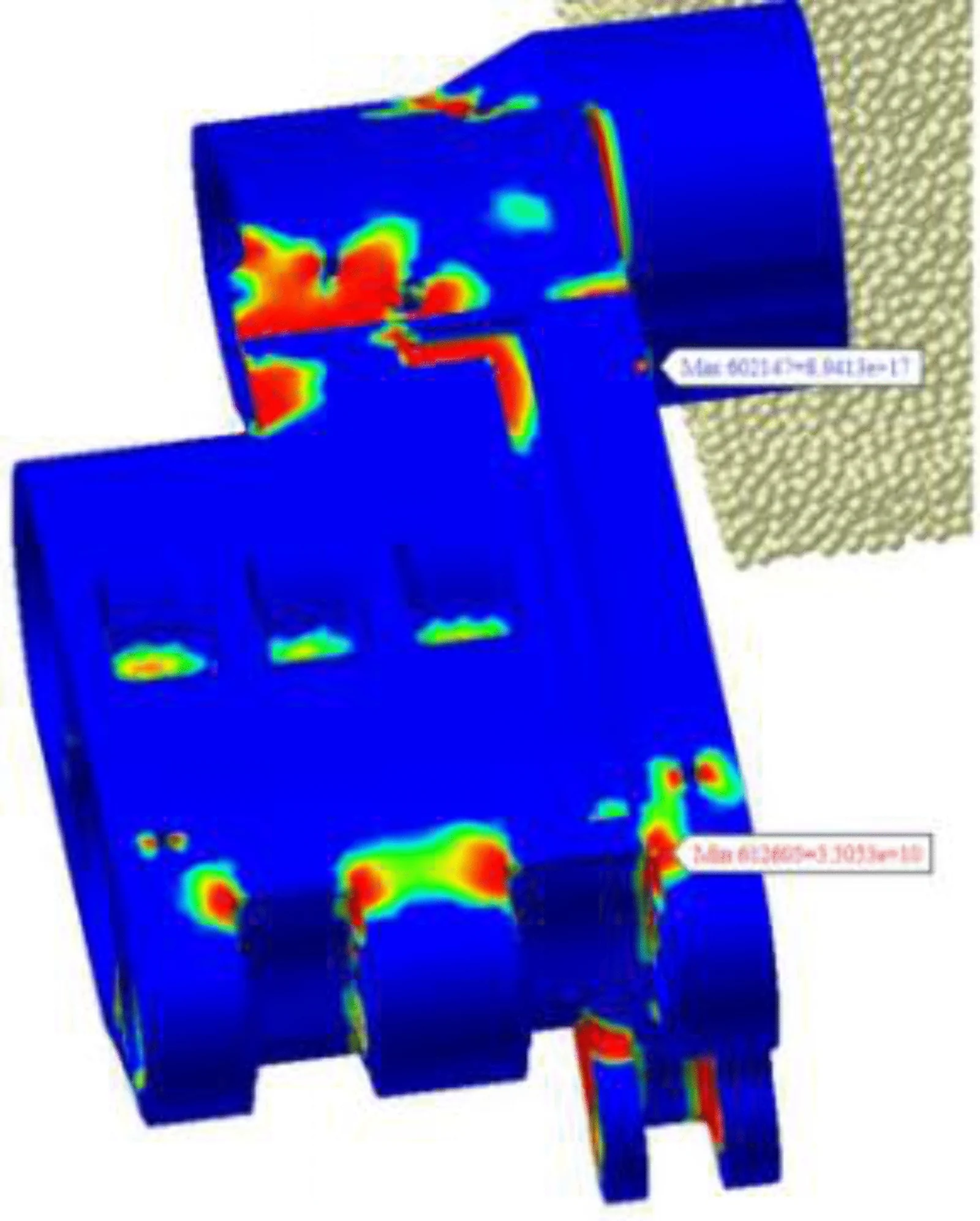
Case fatigue life cloud.

By considering one complete rotation of the spiral drum as the smallest cycle of operation, rainflow counting is performed, and the Palmgren-Miner rule is applied to calculate the cycle count and fatigue life, as shown in the fatigue life cloud diagram in Fig 13.

As shown in Fig 13, the relative fatigue weak zones are concentrated at primary load-transfer and stress-concentration regions, including the housing lugs, drum connection, and gear-shaft holes. These regions transmit drum impact loads to the housing and experience coupled bending and torsion. The minimum contour value occurs near the transition of the upper lug on the coal-cutting side, so the transition radius, weld detail, and local stiffness should be prioritized in design and inspection.

## 4. Conclusion

(1) A DEM-MFBD bidirectional rigid-flexible coupled model was established for a shearer cutting a coal seam containing gangue. The simulated and measured vibration features differ by no more than 4.19%, supporting use of the load spectrum for comparative vibration analysis. Deformation and fatigue outputs remain model-based because they were not independently validated.
(2) Main-effect analysis and ANOVA identify gangue hardness as the dominant factor, contributing 81.68%−82.03% of the maximum, minimum, and RMS responses. Traction speed contributes 8.71%−9.07%, pick arrangement 7.66%−7.74%, drum speed 1.39%−1.49%, and blade angle about 0.11% within the selected ranges. Increasing gangue hardness from 3.5 to 6.8 raises the peak and RMS responses by approximately 35%.
(3) The cross-type pick arrangement reduces maximum and RMS vibration accelerations by 7.73% and 8.57%, respectively, because phase dispersion reduces simultaneous pick impacts. The negative minimum acceleration denotes reverse acceleration along the defined cutting-resistance axis and reflects rebound and intermittent pick disengagement, not negative vibration intensity.
(4) The stress-reliability index identifies Scheme 8 as the most severe condition, with a maximum equivalent stress of 154.73 MPa and an index of 0.9243. Rainflow counting, Palmgren-Miner accumulation, and the Basquin stress-life relation locate fatigue-critical regions at the lug transition, drum connection, and gear-shaft holes. These locations should receive priority in radius optimization, weld-detail control, and maintenance inspection.
(5) The proposed method provides an engineering basis for selecting drum parameters, reducing vibration, and improving cutting unit housing reliability in complex coal seams. The limitations are that deformation and fatigue life were not directly verified by long-duration tests, and interaction effects were not fully separated by the L18 design. Future work should combine field load-spectrum acquisition, mesh-convergence reporting, response-surface interaction analysis, and full-life fatigue validation.

## Supporting information

S1 File Dataset. (XLSX)
